# Sentinel lymph node biopsy for breast cancer patients using fluorescence navigation with indocyanine green

**DOI:** 10.1186/1477-7819-9-157

**Published:** 2011-12-02

**Authors:** Kei Aoyama, Takako Kamio, Tetsuya Ohchi, Masako Nishizawa, Shingo Kameoka

**Affiliations:** 1 Department of Surgery II, Tokyo Women's Medical University, 8-1, Kawada-cho, Shinjuku-ku, Tokyo, 162-8666, Japan

**Keywords:** sentinel lymph node biopsy, breast cancer, indocyanine green, fluorescence imaging

## Abstract

**Background:**

There are various methods for detecting sentinel lymph nodes in breast cancer. Sentinel lymph node biopsy (SLNB) using a vital dye is a convenient and safe, intraoperatively preparative method to assess lymph node status. However, the disadvantage of the dye method is that the success rate of sentinel lymph node detection depend on the surgeon's skills and preoperative mapping of the sentinel lymph node is not feasible. Currently, a vital dye, radioisotope, or a combination of both is used to detect sentinel nodes. Many surgeons have reported successful results using either method. In this study we have analyzed breast lymphatic drainage pathways using indocyanine green (ICG) fluorescence imaging.

**Methods:**

We examined the lymphatic courses, or lymphatic vessels, in the breast using ICG fluorescence imaging, and applied this method to SLNB in patients who underwent their first operative treatment for breast cancer between May 2006 and April 2008. Fluorescence images were obtained using a charge coupled device camera with a cut filter used as a detector, and light emitting diodes at 760 nm as a light source. When ICG was injected into the subareola and periareola, subcutaneous lymphatic vessels from the areola to the axilla became visible by fluorescence within a few minutes. The sentinel lymph node was then dissected with the help of fluorescence imaging navigation.

**Results:**

The detection rate of sentinel nodes was 100%. 0 to 4 states of lymphatic drainage pathways from the areola were observed. The number of sentinel nodes was 3.41 on average.

**Conclusions:**

This method using indocyanine green (ICG) fluorescence imaging may possibly improve the detection rate of sentinel lymph nodes with high sensitivity and compensates for the deficiencies of other methods. The ICG fluorescence imaging technique enables observation of breast lymph vessels running in multiple directions and easily and accurately identification of sentinel lymph nodes. Thus, this technique can be considered useful.

## Background

Sentinel lymph node biopsy (SLNB) is useful to predicts accurate pathological nodal staging and detect the existence of axillary lymph node metastasis. The major problem of axillary dissection is that it produces various morbidities such as serome, infection, numbness and lymphadema of the arm [1,2]. Since April 2010, this technique has been covered by the national health insurance in Japan, promoting its wide use in clinical settings. False-negative of SLNB is a critical problem, as it may lead to inadequate adjuvant therapy, which can negatively affect a patient's prognosis. Currently, three methods are available to identify sentinel lymph nodes: the dye method, the gamma probe method, and a combination of both [3-14]. Although the dye method has several benefits including ease of use, cost effectiveness and safety, it has been pointed out that the detection rate is low compared to the gamma probe method [3-14]. The avoidance of radioisotope is also beneficial especially at institutes where the use of radioisotope is limited. Reports have indicated that the percentage of false-negative SLNB results could be less than 5% in experienced hands if dual-agent techniques are used [3-16]. Meanwhile, the lymphoscintigraphy with a radioisotope cannot clearly visualize the lymphatic drainage pathway. The photodynamic eye (PDE) can visualize the lymphatic drainage pathway clearly and demonstrate the accurate location of sentinel lymph node real-time in the operating room. In our study, we performed SLNB using the indocyanine green (ICG) fluorescence technique to determine whether this can overcome the shortcomings of the dye method, and analyzed breast lymphatic drainage pathways.

## Methods

### Sample collection

Of the 414 patients who underwent their first operative treatment for primary breast cancer between May 2006 and April 2008, 312 patients who were clinically negative for lymph node metastasis were enrolled in this study. A total of 102 patients underwent axillary dissection. Patients whose intraoperative histological results were negative for malignancy underwent no further axillary dissection, whereas the following patients continued to complete axillary dissection: (1) patients with clinically apparent positive nodes (102 cases); (2) patients with positive intraoperative frozen section diagnosis (49 cases,). If the node contained a metastasis, complete axillary dissection was performed immediately (49 cases, 15%).

All patients were informed of the aims of the study, the potential effects of the procedures, the risks associated with surgery, and purpose of randomization. All patients signed a written consent form approved by the institutional ethics committee (institutional review board) of Tokyo Women's Medical University before the operation.

### Operative procedure

SLNB was performed before wide excision, breast conserving surgery, or mastectomy as follows.

**Figure 1:**
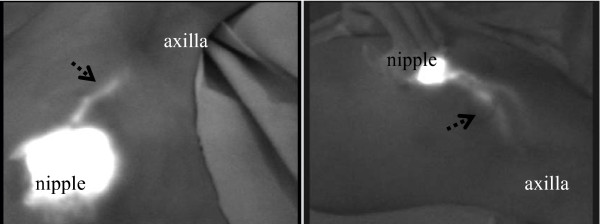
**Two patterns of lympathic drainage pathways from the breast to the axillary basin were found**. (1) One pattern was a subcutaneous lymphatic vessel from the subareolar area running through the anterior surface of the breast into the lymph node in the midaxilla near the intercostals brachial nerve. Patterns of subcutaneous lymphatic drainage pathway from the areola into the axilla: From one to two streams was drained.

**Figure 2:**
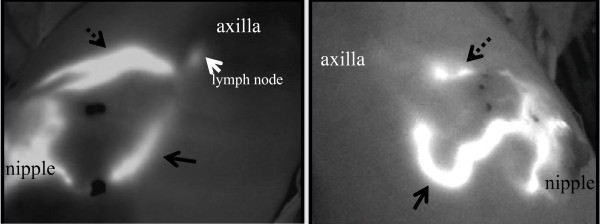
**(2) Another pattern was a lymphatic a lymphatic vessel running directly from the lateral edge of the parenchyma and through the posterior surface of the breast into the lower axilla, and then to the lymph node located at the midaxilla**. Several lymphatic drainage pathways from the areolar area were observed. Several streams from the areola joined together before they drained to the axilla.

**Figure 3:**
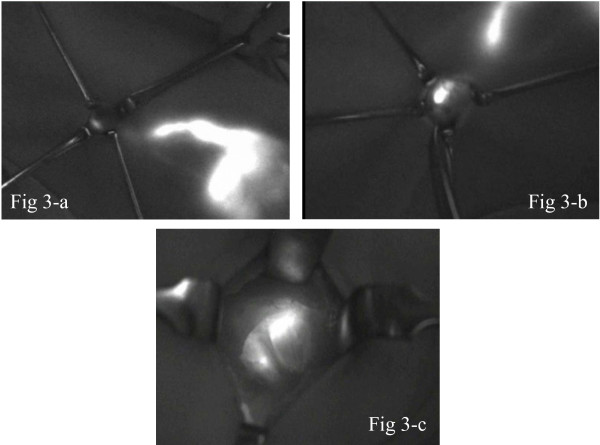
**It is possible to see the fluorescent lymph vessels that run from the body surface to the axilla**. Figure 3-a: A skin incision is made at the end of fluorescence emission that extends to the axilla, in order to observe the fluorescent lymph vessels embedded in the subcutaneous tissue. The fluorescent lymph nodes at the end of the lymph vessels are identified. Figure 3-b: After skin incision, the subcutaneous lymphatics were more clearly visible by fluorescence. Figure 3-c: The lymphatic channels and nodes that received ICG appeared as shining fluorescent streams and spots in the fluorescence image. The lymph node was dissected along with its surrounding fatty tissue.

1) 5 ml of 20 times diluted indocyanine green (ICG) (Diagnogreen 0.5%; Daiichi Pharmaceutical, Tokyo, Japan) is injected into the subareola and periareola. (20 times dilution: ICG is dissolved in 10 ml of solution to be diluted 20 times, i.e., a concentration of 0.125 mg/ml).

2) Breast massage is performed for 10-20 seconds. (ICG is combined with serum proteins and moved into the lymph vessels. Massaging the injected area will increase the interstitial pressure of tissues [17-19], which in turn facilitates the absorption of ICG into the lymph vessels.) Following a few seconds of massage, subcutaneous lymphatic drainage pathway was then observed with fluorescence image (Figure 1, 2).

3) After the surgical lights are turned off, image acquisition is started using the Photodynamic Eye (PDE) infrared camera system manufactured by Hamamatsu Photonics K.K., while infrared light of 760 nm wavelength is being emitted under room fluorescent lighting. (The principle of fluorescence measurement is as follows: Injected ICG molecules are promptly bound to the globulin fractions in serum proteins. Under this condition, when excitation light of 760 nm is irradiated, fluorescence with a peak wavelength of 845 nm will be emitted. This process is captured by a CCD camera with sensitivity in the near-infrared wavelength range).

4) The fluorescence emitted by ICG is followed in the direction from the areola toward the axilla, and a marking is made on the skin where the fluorescence disappears (Figure 1, 2, 3).

5) Skin incision is made at that position and the subdermal layer is removed. Observation by fluorescence imaging is performed again. If there is any site that emits a strong fluorescence, that site is removed along with the surrounding tissue (Figure 3).

6) Lymph nodes in the dissected specimen are isolated from surrounding fatty tissue, and investigated to see whether each lymph node is fluorescent inside. All fluorescent lymph nodes are regarded as sentinel nodes. Anatomical localization of sentinel lymph nodes is recorded according to Berg's level classification. Observation is made again to see if any fluorescence is present on and around the removed lymph nodes (Figure 3).

7) The extracted sentinel lymph nodes are checked for metastasis by intraoperative biopsy with frozen section. Finally, permanent pathological diagnosis is conducted by postoperative HE staining.

All sentinel lymph node positivity were determined intraoperatively by frozen-tissue examination and confirmed postoperatively in formalin-fixed, paraffin-embedded tissue sections stained with hematoxylin and eosin. We did not use immunohistochemistry. Each sentinel lymph node was evaluated at 1 level for nodes of 1 cm or less and at 3 separate levels for nodes of more than 1 cm. Patients given diagnoses of positive sentinel lymph nodes based on frozen-section diagnosis immediately underwent an axillary lymph node dissection of level Ⅰ Ⅱ.

### Statistical analysis

Fisher's exact test was used to evaluate the significance of any relationship between the clinicopathological features and the success of SLNB in identifying sentinel nodes. Differences were considered to be significant when p < 0.05.

## Results

### Identification rate: 100%

The average age was 57.4 years old. Of the 312 patients, 214 had a tumor of less than 2 cm in diameter and 98, a tumor of 2 cm or more, with 95 having had mastectomy and 217 partial mastectomy (Table 1). Non-invasive tumor was found in 47 patients, invasive tumor in 265, and vascular invasion in 152 (Table 2). Hormonal receptor status was positive in 159 patients (50.9%) and negative in 72 (23%). HER-2 status was positive in 46 patients (14.7%) (Table 3).

**Table 1:** Characteristics of patients and tumors

Age: 57.4 (29-85)		
**Diameter of tumor**	< 2.0 cm	214(69%)
	
	> 2.1 cm	98(31%)

**Site of tumor**	Upper inner A	57 (18%)
	
	Lower inner B	24 (8%)
	
	Upper outer C	133 (43%)
	
	Lower outer D	22 (7%)
	
	Central E	76 (24%)

**Operative procedure**	Conservation	95 (30%)
	
	Mastectomy	217 (70%)

**Table 2:** Clinicopathological features

**Histological type**		
A Ductal infiltrating	Intraductal carcinoma	47 (15%)
	Invasive ductal carcinoma	239 (77%)
B Lobular infiltrating		13 (4%)
C Other		13 (4%)
**Peritumoral vascular invasion** **(lymphatic or vascular invasion)**		
Positive		152 (49%)
Negative		160 (51%)

**Table 3:** Tumor characteristics

Hormonal receptor status	ER (+) and PR (+)	159 (51%)
	
	ER (+/-)and/or PR(+/-)	81 (26%)
	
	ER (-) and PR (-)	72 (23%)
**HER2 status**	0	168 (54%)
	
	1+	74 (24%)
	
	2+	24 (7%)
	
	3+	46 (15%)

**Tumor grade**	I	219 (70%)
	
	II	80 (26%)
	
	III	13 (4%)

ER: Estrogen receptor

PR: Progesterone receptor

HER2: Human epidermal growth receptor 2

We observed two main lymphatic drainage pathways draining from the areola in the direction of the axilla: one was directly toward the axilla and the other was toward and through the outside of the mammary gland (Figures 1 and 2). When lymphatic drainage pathway or sentinel lymph nodes were not clearly visible in green color, fluorescence imaging was used for directional guidance. The lymphatic flow through the mammary gland was primarily directed to the axillary lymph nodes, and the flow toward the parasternal lymph nodes or the supraclavicular lymph nodes was not observed in our study.

The number of the lymphatic vessels ranged from one to four, with the average number of identified sentinel lymph nodes being 3.41 (Table 4). (In addition to the fluorescent lymph nodes, the swelling lymph nodes detected by palpation were also sent to intraoperative biopsy as Level I.)

**Table 4:** Number of lymphatic flows and nodes

Direction of lymph current		
(1) Toward the axilla		
(2) Toward and through the outside of the mammary gland		
Number of lymph currents and proportion (%)	0	2%
	
	1	35%
	
	2	46%
	
	3	12%
	
	4	5%

Identification rate: 100%		

Identified lymph nodes (average)	Sentinel lymph nodes	3.41 (range: 1-12)
	
	Level I	1.66 (range: 0-10)
	
	Total	5.07 (range: 1-17)

Patients whose intraoperative histological results were negative for malignancy underwent no further axillary dissection, whereas the following patients continued to complete axillary dissection: (1) patients with clinically apparent positive nodes (102 cases); (2) patients with positive operative frozen section diagnosis (49 cases). When the presence of lymph node metastasis was observed in intraoperative biopsy, a total dissection of the axillary lymph nodes was performed (49 patients, 15%). There were 8 patients in whom metastatic lesions were not found in the identified lymph nodes by intraoperative biopsy but metastasis was detected in permanent preparation at a later date. These were false negative cases, resulted from the fact that metastatic lesions in the slice for intraoperative biopsy were too small and it was only possible to detect such lesions using permanent preparation. Any metastatic lesion in the 8 patients who showed false negative results for metastasis was very small, less than 2 mm, which prevented detection by intraoperative biopsy. Side effects such as intraoperative allergy due to ICG were not observed.

Of the 263 patients who showed negative results for sentinel lymph node metastasis, 6 patients developed postoperative distant metastasis (supraclavicular lymph nodes in one patient, subpectoral lymph nodes in one, lung in two, brain in one and liver in one). Of the 49 patients in which the presence of sentinel lymph node metastasis was observed by intraoperative biopsy and a total dissection of the axillary lymph nodes was performed, 4 patients developed postoperative distant metastasis (parasternal lymph nodes in one patient, lung in one, bone in one and brain in one) (Table 5). None of the patients in this study suffered disease relapse in axillary lymph nodes during a median follow-up period of 49 months.

**Table 5:** Unfavorable events and deaths

	SLNB Group (n = 263)	SLNB- > ALND Group (n = 49)
Unfavorable events	0	0

Events other than death	0	0

Axillary metastasis	0	0

Supraclavicular metastasis	1 (0.3%)	0

Recurrence in ipsilateral breast	18 (0.3%)	0

Cancer in contralateral breast	0	0

Distant metastasis	5 (2%)	4 (8%)

Other primary tumor	0	0

Death due to breast cancer	3 (1%)	2 (4%)

Death from other causes	0	0

ALND: Axillary lymph node dissection

SLNB: Sentinel lymph node biopsy

SLNB- > ALND: Because of being positive for lymph node metastasis in sentinel lymph node biopsy, a total dissection of the axillary lymph nodes was applied.

Statistical significance was evaluated using the chi-square test or Fisher's exact probability test. There were no significant differences between the two groups. (P = 0.55)

Of the patients who received sentinel lymph node biopsy, 1 developed metastasis in the remaining breast after breast conserving surgery. In those who tested negative for metastasis by SLNB, no patient developed metastasis in the axillary lymph nodes (Table 5).

## Discussion

Many studies have been conducted on SLNB, since the publication by Krag et al. on the method using radioisotope in 1993 [10,11], and a report by Giuliano et al. on the dye method in 1994 [12,13]. Veronesi et al suggested the possibility that a total dissection of the axillary lymph nodes is not always necessary, and a large scale RCT was carried out [1,2,20-29]. These reported successful lymphatic mapping in 92% using a combination of a dye and a gamma probe method. They found that SLNB using dye alone allowed the identification in approximately 70-80% of patients and that adding a radioisotope improved the detection rate. It is therefore certain that the combination of dye and radioisotope will become more commonly used in SLNB [3-16].

It is important, therefore, that the accuracy of SLNB be secured by each surgeon and each institute. Currently, two types of tracers, dye and radioisotope, are used to detect the sentinel lymph nodes in breast cancer patients, and a combined use of the two tracers has yielded a high diagnostic value [3-14].

We performed the SLNB using the ICG fluorescence measurement technique in order to overcome some drawbacks inherent in the dye method (Table 6). At our department, the identification rate of sentinel lymph nodes is 100%. This is a result similar to or better than those reported by other researchers, which were obtained by the gamma probe method or the combined method that uses the gamma probe and the dye [3-14]. Thus, it can be said that SLNB using ICG fluorescence could be very useful.

**Table 6:** Benefits and Drawbacks of ICG Flourescence Method

Benefits of ICG fluorescence method	Drawbacks of ICG fluorescence method
(1) The subcutaneous lymphatic vessels running from the areola to the axilla can be observed from outside, through the skin allowing of the accurate determination location of the skin incision in the axilla.	(1) If lymph vessels that have been emitting fluorescence signal are damaged, ICG will leak into the surrounding tissue, preventing an accurate identification of sentinel lymph nodes.

(2) Fluorescence can be used as a guide to remove the lymph vessels running toward the axilla more easily.	(2) Many lymph nodes that are emitting fluorescence, with an average of five or more, can appear

(3) If any fluorescence signal is detected in the extracted lymph nodes, it means there are sentinel lymph nodes in those lymph nodes.	(3) Skin pigmentation due to ICG remains for a certain period of time.

(4) Other benefits include: no exposure to radiation, easy to use, and cost effectiveness.	

We usually inject ICG into the subareola and periareola. Because the mammary gland is embryologically derived from the ectoderm, it is thought that the mammary gland tissue and the subcutaneous tissue share common lymphatic vessels, which lead to the relevant lymph nodes [30-34]. Lymphatic flow originating in the mammary gland tissues first moves into the subareolar lymphatic vessels, and then drains to the axillary lymph nodes. Therefore, the injection of ICG into the subareola and periareola allows us to identify the same sentinel lymph nodes as those identified with the peritumonal injection. Some studies [30-34] attempted to clarify whether injection of mapping agents into the subareolar breast tissue results in the identification of the same sentinel lymph nodes as injection into intradermal sites overlying the breast tumor. It would be simpler if the injection could be performed in the same manner for all patients regardless of tumor location or palpability. Other studies [30-34] have shown that a dermal or subdermal injection of mapping agents results in more reliable sentinel lymph node identification than a peritumonal injection. It has been hypothesized that the lymphatic drainage pathway of the entire breast flows to the same few sentinel lymph nodes. No lymphatic drainage pathway was observed to the parasternal lymph node and the supraclavicular lymph node. It was reported that the lymphatic drainage pathway in the breast parenchyma flowed toward the subareolar lymphatic plexus and drained in to the axilla [30-34]. In the present study, a lymphatic drainage pathway from the areolar area was detected in almost all patients.

In our study, ICG, which was injected into the subareola and periareola, emitted a high level of fluorescence at the injection location, then promptly moved into the lymphatic vessels, and finally spread further through the lymphatic flow courses. The PDE infrared camera system has an ability to capture fluorescence up to a depth of 1 cm from the skin surface. In patients with a low body mass index (BMI), it was possible to directly observe the fluorescent lymph nodes in the axillary region because their subcutaneous fat layer was thin.

On the other hand, patients with a high BMI usually have a thick subcutaneous fat layer, making it difficult or impossible to track the lymphatic drainage pathway toward the axilla by fluorescence. It seems that in 7 patients (2%) failure to detect a lymphatic drainage pathway was unavoidable, as all 7 patients were obese (BMI > 30). BMI has been shown to correlate with an increased incidence of failure in identifying a sentinel lymph node. The specific causes of mapping failure remain uncertain, but some contributing factors have been observed [35-39]. Using inadequate volume of injection, inadequate dispersion, inappropriate timing, and inadequate massage may weaken the ability of the dyes and radioisotopes to reach the nodal basin. Fatty tissue around the lymph nodes may also cause decreased flow to the lymphatic basin. Successfully lymphatic drainage pathway mapped patients had a mean BMI of 22.3 kg/m^2 ^(range 17.9 to 33 kg/m^2^).

However, for these obesity patients (BMI > 30), it was possible to easily identify the lymph nodes and lymphatic vessels embedded in the adipose tissue by incising the subcutaneous fat in the axillary incision site. The intensity of fluorescence can be adjusted if required. It is also possible to distinguish between the fluorescence from the lymph nodes or lymphatic vessels and the fluorescence from ICG that happens to attach somewhere. If the fluorescence is strong, and can determine whether the lymph nodes or lymphatic vessels, the deposit of a ICG is also possible to adjust the high and low emission intensity of the device. None of these obesity 7 patients were not failed to identify sentinel lymph node and not had false negative sentinel lymph nodes.

More sentinel lymph nodes were detected by ICG fluorescence imaging compared with other methods (Table 4) [1-14,17,18]. The uptake of the localization agents into the lymphatic vessels is dependent on the interstitial pressure [19,30-39]. Our data from past experiences suggest that ICG fluorescence could detect a wide range of sentinel lymph nodes. ICG in the lymph node can be illuminated by fluorescence with very high sensitivity even if it does not appear green. When we detect many harvested sentinel lymph nodes, the following can be suggested according to several differences between the dye and radioisotope: (1) the molecular weight and the chemical structure, (2) the intensity of binding of some kinds of protein to the tracers, (3) the injection time to tracers, and (4) the sensitivity of methods [3,4]. The small blue dye crystals passively rely on prevailing fluid dynamics for visualization of the lymph node as fluorescence is emitted. Our experiments clearly revealed that extensive sentinel lymph nodes in the axilla could be detected only with the ICG fluorescence method. In our department, 20 times diluted ICG is used. The intensity level of fluorescence obtained from this ICG is appropriate, allowing us to perform the SLNB in an effective and efficient manner. It was possible to identify the irregularities, which may appear around the fluorescence emission when some lymphatic vessels are damaged, by adjusting the emission intensity of fluorescence. It is also possible to distinguish the lymph node from the lymphatic vessel by adjusting the fluorescence emission intensity. (The lymph nodes illuminate in a spotty pattern, whereas the lymph vessels illuminate in a linear or streaky pattern.)

We observed two main lymphatic drainage pathways draining from the areola in the direction of the axilla: one was directly toward the axilla and the other was toward and through the outside of the mammary gland. Regarding the number of lymphatic vessels, it was observed in many patients that several lymphatic vessels running in the same direction join together to form the lymphatic vessel network. In some cases, it flows into multiple sentinel lymph nodes through multiple lymphatic vessels (Figure 1, 2).

When the presence of lymph node metastasis was observed in intraoperative biopsy, a total dissection of the axillary lymph nodes was performed. However, additional surgery is not usually performed for patients in which micrometastasis is detected by the postoperative HE staining of permanent preparation but other lymph nodes removed as part of a group sentinel are metastasis-negative. Currently, postoperative tumor recurrence in the axillary lymph nodes has not been observed (Table 5). The clinical significance of micrometastasis and ITC (isolated tumor cell) has not been established. These were false negative cases, resulting from the fact that metastatic lesions in the slice for intraoperative biopsy were too small and it was only possible to detect such lesions using permanent preparation. Any metastatic legion in the 8 patients who showed false negative results for metastasis was very small, less than 2 mm, which prevented detection by intraoperative biopsy. However, some reports have suggested that there might be an association between micrometastasies in the lymph nodes and distant metastasis [27-29]. The prognostic significance of a micrometastasis in terms of metastatic risk is controversial [1,2,20-29].

At present, no standard guidelines are available for dealing with micrometastasies in the sentinel lymph nodes. Among the patients who underwent a total dissection because metastasis had been detected in a single sentinel lymph node, no patient developed metastasis in other organs or parts other than the sentinel lymph nodes. If we are able to accurately identify the patients with metastasis only in the sentinel lymph node(s), it would be possible to reduce the need for further surgery or unnecessary total dissections.

## Conclusions

The fluorescence method allows us to see how the lymphatic drainage pathway flows from the areola toward other parts in real-time during surgery, as well as clearly understand how the lymphatic vessels run from the areola to the axilla. With the dye method, sometimes it is difficult or impossible to visually determine whether the extracted lymph nodes are sentinel lymph nodes or not. Meanwhile, with the fluorescence method, this can be done relatively easily by checking the existence of fluorescence in the extracted lymph nodes. This method is a technique that enables us to easily identify the sentinel lymph nodes during surgery with low invasiveness and no exposure to radiation, thus providing a safe and very useful way to detect the axillary sentinel lymph nodes in patients with breast cancer, in additon to improving the identification rate.

## Abbreviations

(ICG): indocyanine green; (CCD): charge coupled device; (LED): light emitting diode; (SLN): sentinel lymph node; (SLNB): sentinel lymph node biopsy; (ALND): axillary lymph node dissection; (ER): estrogen receptor; (PR): progesterone receptor; (HER2): human epidermal growth receptor 2; (BMI): body mass index; (RI): radioisotope; (ITC): isolated tumor cell.

## Competing interests

The authors declare that they have no competing interests.

## Authors' contributions

KA designed the study, researched the literature, and drafted the manuscript. TK, TO, MN, and SK participated in the study design and coordination, and helped to collect data. All authors have read and approved the manuscript.
